# Andrographolide Induces Apoptosis of C6 Glioma Cells via the ERK-p53-Caspase 7-PARP Pathway

**DOI:** 10.1155/2014/312847

**Published:** 2014-08-05

**Authors:** Shih-Hung Yang, Seu-Mei Wang, Jhih-Pu Syu, Ying Chen, Sheng-De Wang, Yu-Sen Peng, Meng-Fai Kuo, Hsiu-Ni Kung

**Affiliations:** ^1^Division of Neurosurgery, Department of Surgery, National Taiwan University Hospital, No. 7, Zhongshan South Road, Zhongzheng District, Taipei City 100, Taiwan; ^2^Department of Anatomy and Cell Biology, College of Medicine, National Taiwan University, 1-1 Jen-Ai Road, Taipei 10051, Taiwan; ^3^Department of Biology and Anatomy, National Defense Medical Center, No. 161, Section 6, Minquan East Road, Neihu District, Taipei City 114, Taiwan; ^4^Division of Nephrology, Department of Internal Medicine, Far Eastern Memorial Hospital, No. 21, Section 2, Nanya South Road, Banqiao District, New Taipei City 220, Taiwan

## Abstract

*Background.* Glioma is the most malignant tumor of the central nervous system. Efforts on the development of new chemotherapy are mandatory. Andrographolide (AND), a diterpenoid lactone isolated from the *Andrographis paniculata*, has been shown to have antitumor activities in several types of cancer cells. Whether AND can exert its antitumor activity in glioblastoma cells remains unknown. This study examined the anticancer effects of AND, both *in vitro* and *in vivo*. *Methods.* Cell apoptosis was assayed by flow cytometry and nuclear staining. The signaling pathway for AND was determined by western blotting. The effects of AND on tumor growth was evaluated in a mouse model. *Results and Conclusion. In vitro*, with application of specific inhibitors and siRNA, AND-induced apoptosis was proven through ROS-ERK-P53-caspase 7-PARP signaling pathway. *In vivo*, AND significantly retarded tumor growth and caused regression of well-formed tumors *in vivo*. Furthermore, AND did not induce apoptosis or activate ERK and p53 in primary cultured astrocyte cells, and it may serve as a potential therapeutic candidate for the treatment of glioma.

## 1. Introduction

Glioma is the most common malignant tumor of the central nervous system [1]. These tumors, including astrocytoma, oligodendrogliomas, ependymomas, and other rare types of glial tumors, arise from glial cells. Due to their infiltrative nature and frequent involvement of eloquent regions in brain and spinal cord, surgical removal is usually not possible. These patients often need to control their diseases through adjuvant therapies such as radiotherapy and chemotherapy. Other therapeutic agents against specific targets, including antivascular endothelial growth factor (VEGF) monoclonal antibody (bevacizumab) and epidermal growth factor receptor (EGFR) inhibitors, are also being used for disease control in glioma [2, 3]. However, failure of treatment inevitably occurs. Among all kinds of glioma, glioblastoma, which is associated with extremely poor prognosis, is the most frequent and malignant type of glioma. The 2-year survival rate is 7.5%, and 5-year survival rate reduced to only 5% [4, 5]. Most patients die of glioblastoma within 2 years. Therefore, scientists and clinicians worldwide are still searching for better therapies for malignant gliomas.

Andrographolide (AND) is a diterpenoid lactone molecule that possesses various biological activities, including anti-inflammatory [6], immunomodulatory [7], hepatoprotective [8], antiviral [9], and antitumoral effects [10]. It is extracted from the stem and leaves of the medicinal plant,* Andrographis paniculata*. AND treatment blocked the* in vitro* proliferation of a variety of tumor cell lines, such as neuroblastoma, melanoma, hepatoma, prostate cancer, and gastric cancer [11–14]. This compound exerts anticancer activity on tumor cells by several mechanisms, such as cell-cycle arrest [13], growth factor signaling modulation, cellular migration [15], and angiogenesis. For example, AND inhibited the growth of colorectal carcinoma LoVo cells by inducing expression of p53, p21, and p16, resulting in repression of Cyclin D/Cdk4 and/or Cyclin E/Cdk2 activities, as well as Rb phosphorylation, thus leading to G1-S phase arrest [16]. AND also inhibits human hepatoma Hep3B cell growth through JNK activation [17]. In epidermoid carcinoma cells, AND decreased cell proliferation through enhanced degradation of EGFRs on the cell surface [18]. It also inhibited migration of colorectal carcinoma LoVo cells and non small cell lung cancer A549 cells by suppression of PI3K/Akt signaling pathway, which decreased the mRNA and protein levels of matrix metalloproteinase-7 (MMP-7) [19, 20]. Furthermore, AND reduced VEGF level in both B16F-10 melanoma cells and A549 lung cancer cells [21, 22], which blocked angiogenesis around tumors. In addition, AND induces cell death in various tumor cell types. In HL-60 leukemic cells, AND treatment resulted in disappearance of mitochondrial cytochrome C, increased expression of Bax, and decreased expression level of Bcl-2 proteins [23]. In B16F-10 melanoma cells, AND modulated p53-induced-caspase-3 expression [24]. A recent study demonstrated that AND inhibited cell proliferation via inactivation of PI3K/AKT signaling in human glioblastoma cells [25]. Beside, AND also sensitizes cancer cells to TRAIL-induced apoptosis via p53 [26]. Whether AND induces programmed cell death (apoptosis) in glioma cells and the mechanisms underlying AND-induced cell death remain to be determined.

In this report, we aimed to study the antitumor effects of AND on C6 glioma cells, which is an experimental model of glioblastoma [27], and the underlying mechanisms.

## 2. Materials and Methods

### 2.1. Cell Culture

C6 glioma cells, a rat cell line of astrocytic origin, were purchased from the American Type Culture Collection (Rockville, MD, USA). The primary rat astrocyte cell line was a generous gift from Dr. Jiahn-Chun Wu (National Yang-Ming University, Taiwan) [28]. The cells were grown in Dulbecco's modified Eagle's medium (DMEM) containing 10% fetal bovine serum (both from Gibco BRL, Grand Island, NY), 1 mM sodium pyruvate (Sigma, St. Louis, MO, USA), and 100 IU/mL penicillin and streptomycin (pH 7.2) (Gibco BRL, Grand Island, NY). Cells were incubated in a humidified atmosphere of 5% CO_2_/95% air at 37°C.

### 2.2. Drugs

AND, propidium iodide (PI), and 4,6-diamidino-2-phenylindole dilactate (DAPI) were purchased from Sigma. 3AB, Z-VAD, and DEVD were purchased from Biomol (Enzo Life Sciences Inc., NY, USA). PD98059 was purchased from Cell Signaling Technology Inc. (Beverly, MA, USA).

### 2.3. Cell Survival Assay

Cells were plated at 8 × 10^3^ cells per well of a 24-well plate and incubated for 24 h for cell adhesion. Different concentrations of AND or 0.2% dimethyl sulfoxide (DMSO, Sigma) were added to the culture medium for 12 or 24 h as indicated. After washing twice with phosphate-buffered saline (PBS) (137 mM NaCl, 2.7 mM KCl, 1.5 Mm KH_2_PO_4_, and 8 mM Na_2_HPO_4_, pH 7.4), 0.5 mL of DMEM medium containing 0.5 mg/mL of 2.3.3-(4,5-dimethylthiazol-2-yl)-2,5-diphenyltetrazolium bromide (MTT) (Sigma) was added to each well and incubation was continued for another 2 h. The reaction solution was then removed, and the cells were lysed with 0.5 mL of DMSO and the absorbance at 590 nm was determined using a spectrophotometer (Beckman Coulter Inc., Fullerton, CA, USA).

### 2.4. Apoptosis Detection Assays

For detection of apoptosis, two methods were used in the study. First, cells were treated with AND for 0–24 h and then trypsinized. After washing with cold PBS, the cells were stained with Apoptosis Detection kit (Strong Biotech Corporation, AVK050, Taipei, Taiwan), containing identified annexin V-FITC and PI in 100 *μ*L of binding buffer, for 15 min and analyzed by flow cytometry. FL1 and FL2 represented the intensity of FITC and PI, respectively. DAPI stain was also used to detect the apoptotic process in cells. Cells were seeded on the cover slides. After various treatments, cells were washed with ice cold PBS and stained for 15 min with 1 *μ*g/mL DAPI in 0.9% NaCl. Cover slides were mounted on the slides using fluorescence mounting medium (70% glycerol and 2% propyl gallate in PBS). Cell images were captured using a fluorescence microscope and a digital camera.

### 2.5. Small Interfering RNA (siRNA) Transfection

A siRNA for p53, which targeted the RNA coding sequence, was designed by Dharmacon (ON-TARGET plus SMARTpool, Dharmacon Corporation, Lafayette, CO, USA). Negative control and GAPDH siRNAs were purchased from Ambion (Silencer Select Predesigned siRNA, Ambion, Austin, TX, USA). The siRNAs were transfected through electroporation, as specified in the instruction manual (Amaxa, Germany). After transfection, cells were cultured for 48 h to detect target expression. Briefly, 10^6^ cells were trypsinized and resuspended in 100 *μ*L of Nucleofector solution (Amaxa), and 100 nM of siRNA duplexes was electroporated.

### 2.6. Western Blotting

After the various treatments, cells were washed once with ice cold PBS, homogenized in lysis buffer (10 mM EGTA, 2 mM MgCl_2_, 60 mM PIPES, 25 mM HEPES, 0.15% triton X-100, 1 *μ*g/mL pepstatin A, 1 *μ*g/mL leupeptin, 1 mM NaF, and 1 mM phenylmethylsulfonyl fluoride) and sonicated twice for 10 s each time. The concentrations of proteins were determined using a Bio-Rad Protein Assay kit (Bio-Rad Life Science, Hercules, CA, USA), and samples of proteins (80 or 120 *μ*g per lane) were electrophoresed on a 10% SDS polyacrylamide gel and transferred to a nitrocellulose membrane (Schleicher & Schuell Inc., Keene, NH, USA). Strips from the membrane were then blocked by incubation with 5% nonfat milk in Tris-buffered saline (pH 8.2, containing 0.1% Tween (TBS-Tween)) for 1 h at room temperature and then incubated overnight at 4°C with a 1 : 5000 dilution of monoclonal rabbit antibody against GAPDH (GeneTex Inc., Irvine, USA), 1 : 500 dilution of phosphor-extracellular-signal-regulated kinases (ERK) or phospho-P38 (Santa Cruz Biotechnology, Inc., California, USA). Other blots were incubated with a 1 : 500 dilution of monoclonal rabbit antibodies against caspase 3, cleaved caspase 3, caspase 7, cleaved caspase 7, cleaved poly (ADP-ribose) polymerase (PARP), p53, phospho-p53 (Ser15), or phospho-c-Jun *N*-terminal protein kinase (phospho-JNK) (Cell Signaling Technology, Inc., Beverly, MA, USA), all diluted in TBS-Tween. After washing with TBS-Tween, the strips were incubated for 2 h at room temperature with a 1 : 7500 dilution of alkaline phosphatase-conjugated anti-mouse or anti-rabbit IgG antibodies (Promega Corp., Madison, WI, USA), and the bound antibody was visualized using nitro blue tetrazolium and 5-bromo-4-chloro-3-indolyl phosphate (Sigma) as a chromogen. The density of the bands on the nitrocellulose membrane was quantified by densitometry using Gel Pro 3.1 (Media Cybernetics, Silver Spring, MD, USA), setting the density of the band in the control sample as 100% and expressing the density of the band in the test sample as a percentage of the control band density.

### 2.7. Animals

Adult ICR male mice (8-week old) were purchased from the National Taiwan University Animal Center and housed in individual cages in a temperature- and humidity-controlled room (12 : 12 h light-dark cycle) with free access to tap water and diet. All of the animal experiments were performed according to National Institutes of Health guidelines and were approved by the Laboratory Animal Committee of the College of Medicine, National Taiwan University.

### 2.8. *In Vivo* Experiment

The* in vivo* tumor growth model in the ear was performed according to previous studies [29–32] with some modifications. Two kinds of* in vivo* experiments were performed, coinjection or postimplantation AND injection. First, the ears of 8-week-old male ICR mice were subcutaneously injected in the center with 1 × 10^7^ C6 cells with (right ear) or without (left ear) 20 *μ*M AND. The ears were photographed under a dissecting microscope at day 5 after injection. The tumor tissues were weighted and photographed, and the results were expressed as a relative percentage of that of the control side (left ear). Second, in the postimplantation AND injection experiment, 1 × 10^7^ C6 cells were injected in the middle of both ears in ICR mice. Pictures of tumors were taken at day 3. 30 *μ*L of saline (left ear) or 20 *μ*M AND (right ear) was injected into the tumors twice at day 3 and day 6. The tumor tissues were removed from ears at day 9, weighted, and pictured. The weight of tumor tissues was calculated by microbalance, and take left tissue volume as 100%.

### 2.9. Statistical Analysis

All experiments were performed at least 3 times, and the results are expressed as the mean ± SEM for the total number of experiments. We assessed statistical differences between means by using one-way ANOVA test and posttested them using Dunnett's test. A *P* value of less than 0.05 was considered statistically significant ( * or  ^#^), and a value of less than 0.01 was considered more statistically significant ( **).  *: compared to CTL group,  ^#^: compared to AND group.

## 3. Results

### 3.1. AND Induced Cell Death of C6 Glioma Cell by Apoptosis

**Figure 1:**
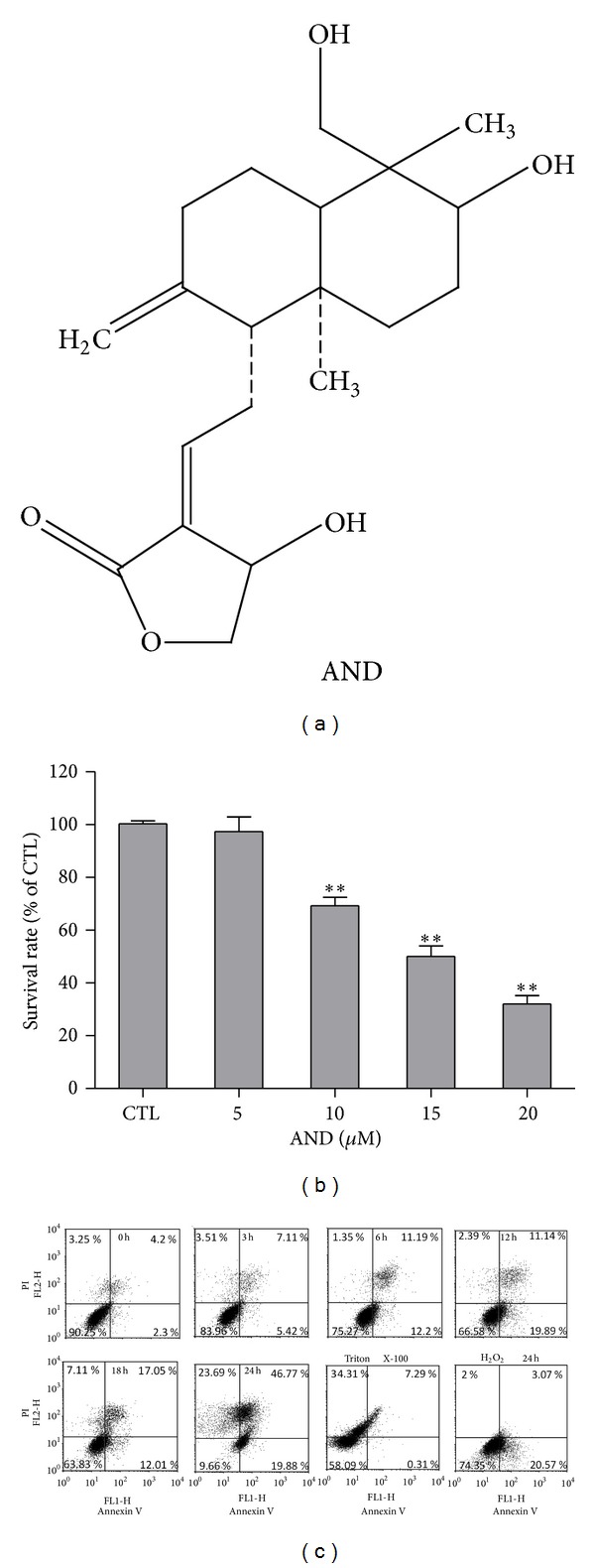
The structure of AND and the effect of AND on the survival of C6 glioma cells. (a) The chemical structure of AND. (b) The cells were treated with 0.1% dimethyl sulfoxide (DMSO) (CTL), 5, 10, 15, or 20 *μ*M of AND for 24 h, and cell viability was determined using the MTT assay. *N* = 3. **P < 0.01, compared to the control group. (c) Flow cytometric analysis of AND-induced apoptosis. Cells were treated with 15 *μ*M AND for different intervals and stained with annexin V and propidium iodide PI for flow cytometric analysis.

**Figure 2:**
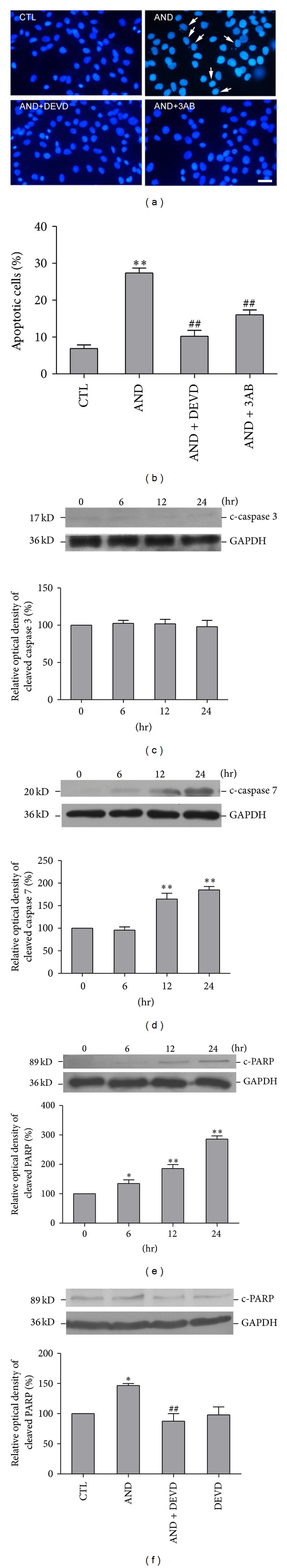
The apoptotic effects of AND on C6 glioma cells, and the involved signaling molecules. (a) 4,6-Diamidino-2-phenylindole dilactate (DAPI) staining. The cells were treated with 0.1% DMSO (CTL), 15 *μ*M AND, 15 *μ*M AND plus 50 *μ*M DEVD, and 15 *μ*M AND plus 5 *µ*g/mL 3AB for 12 h and stained with DAPI. Apoptotic nuclei (arrowheads) were identified by nuclear morphology. Bar = 20 *μ*m. (b) Quantitative data from (a). *N* = 7. *P < 0.05, **P < 0.01, compared to the control group. ^##^ *P* < 0.01, as compared to the AND group. ((c)–(e)) The protein expression levels of cleaved caspase (c-caspase) 3 (c), c-caspase 7 (d), and cleaved PARP (c-PARP) (e). Cells were treated with 15 *μ*M AND for 0, 6, 12, or 24 h, and cell lysates were analyzed for target proteins and GADPH (internal standard). *N* = 3. (f) Effects of DEVD. The cells were treated with 0.1% DMSO, 15 *μ*M AND, 15 *μ*M AND plus 50 *μ*M DEVD, or 50 *μ*M DEVD for 24 h, and cell lysates were analyzed for cleaved PARP and GADPH. *N* = 3. *P < 0.05, **P < 0.01, as compared to the control group. ^##^ *P* < 0.01 as compared to the AND group.

The chemical structure of AND is shown in Figure 1(a). C6 glioma cells were treated with various concentrations of AND for 24 h, and cell viability was analyzed by MTT assay (Figure 1(b)). The effect of AND glioma cell survival was found to be dose-dependent. Compared to cells treated with DMSO (control group), cells treated with 5 *µ*M AND showed either no survival benefit or no toxic effect. The cell survival rate of cells treated with 10 to 20 *µ*M of AND decreased from 70% to 30%, and the IC50 of AND was approximately 15 *µ*M. Therefore, 15 *µ*M of AND was used in the subsequent time-dependent experiments. Following treatment with DMSO or 15 *µ*M of AND for different intervals, C6 glioma cells were stained by annexin V and PI or DAPI for analyzing the cell death pattern. As determined by flow cytometry, the proportion of apoptotic cell with annexin V labeling increased with time. The cell population shift from negative stain (Figure 1(c), left down square) to annexin V-positive (Figure 1(c), right down square), and double positive (Figure 1(c), right up square) sequentially defined that AND induced cell death by most apoptosis (Figure 1(c)). DAPI staining identified apoptotic cells by the presence of apoptotic nuclei (Figure 2, arrows). The results revealed that there were very few apoptotic cells in the DMSO group but significant number of apoptotic cells in the AND groups. The percentage of apoptotic cells was 6.7% ± 1.6% in the DMSO group and 28.9% ± 1.6% in the AND group (15 *μ*M, 12 h).

### 3.2. AND Triggered Caspase 7-PARP Signaling in C6 Glioma Cells

To delineate the signal transduction pathway of apoptosis, DEVD (5 *µ*g/mL, caspase 3/7 inhibitor) or 3AB (5 *µ*g/mL, PARP inhibitor) was used for 30 min before AND treatment. Pretreatment of C6 cells with DEVD or 3AB inhibited AND-induced apoptosis, and the percentages of apoptotic cells were 7.8% ± 1.3% and 15.8% ± 2.0%, respectively, which were significant compared to AND alone (Figure 2). MTT assay and annexin V binding assay were performed to further investigate whether caspase 7 and PARP were involved in AND-induced cell death. Both inhibitors blocked the cytotoxicity of AND (see Figure 1 in Supplementary Material available online at http://dx.doi.org/10.1155/2014/312847). These findings indicated that AND-induced cell death was caspase 3/7- and PARP-dependent.

Because the caspase 3/7 inhibitor, DEVD, effectively blocked AND-induced apoptosis, we further analyzed the role of caspase 3/7 in the apoptotic pathway. Several activated caspases are self-cleaved into 2 subunits, permitting identification of the activation of caspase by the presence of cleaved caspase (c-caspase). Following AND treatment, the levels of c-caspase 3 in C6 cells did not change significantly in comparison to DMSO treatment (Figure 2(c)), but c-caspase 7 levels increased significantly, and this increase showed both a dose-dependent (Supplementary Figure 2(a)) and a time-dependent trend (Figure 2(d)). The protein levels of c-caspase 7, following treatment with 20 *µ*M of AND for 12 and 24 h, increased to 1.8- and 2.2-fold, respectively (Figure 2(d)). These results suggest that AND induced caspase 7 activation.

Once activated, caspase 7 cleaves many of the same substrates as caspase 3, including poly (ADP-ribose) polymerase or PARP [33, 34]. Activation of caspase 3 or 7 results in cleavage of the downstream protein PARP, which is an excellent marker for apoptosis [35]. Like caspases, activated PARP is self-cleaved into 2 subunits, permitting the activation of PARP to be identified. With the PARP inhibitor, 3AB, which effectively blocked AND-induced apoptosis (Figures 2(a) and 2(b)), we further analyzed the role of PARP in the apoptotic pathway. Following AND treatment, the levels of cleaved PARP (c-PARP) in C6 cells increase significantly and showed a dose-dependent (Supplementary Figure 2(b)) as well as a time-dependent trend (Figure 2(e)). Quantitative analysis showed that treatment with AND for 24 h at concentrations of 10 *µ*M, 15 *µ*M, and 20 *µ*M induced c-PARP to 1.5-, 3.5-, and 3.8-fold, respectively (Supplementary Figure 2(b)). Treatment with 15 *µ*M AND for 12 h and 24 h elevated the levels of cleaved PARP to 1.9- and 2.9-fold, respectively (Figure 2(e)). Pretreatment with the caspase 3/7 inhibitor, DEVD, blocked the AND-induced elevation of c-PARP levels (Figure 2(f)). Therefore, AND induced apoptosis via the caspase 7-PARP signaling pathway.

### 3.3. AND Increased the Expression of p53 and Activated p53

**Figure 3:**
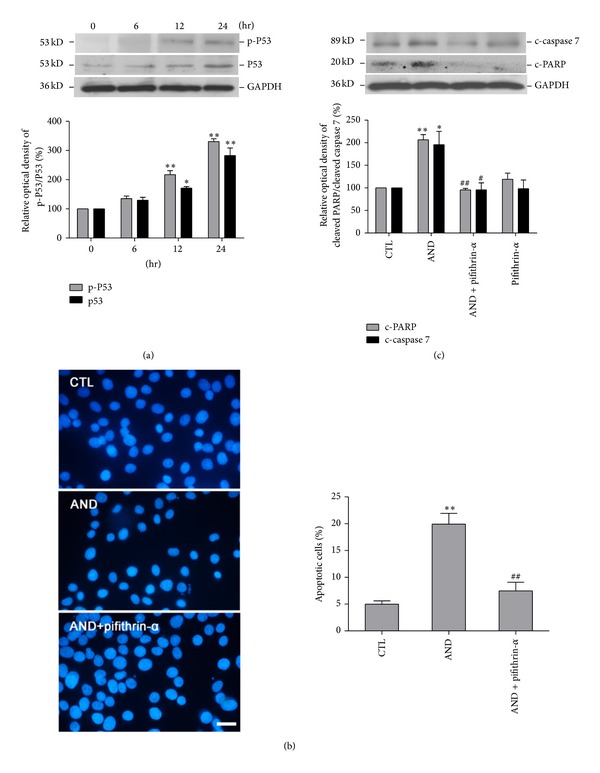
p53 and its downstream molecules were involved in AND-induced apoptosis in C6 glioma cells. (a) The expression of p-p53 and p53. Cells were treated with 15 *μ*M AND for 0, 6, 12, or 24 h, and cell lysates were analyzed for total p53 and p-p53. *N* = 3. (b) DAPI stain. Cells were treated with 0.1% DMSO, 15 *μ*M AND, or 15 *μ*M AND plus 15 *μ*M pifithrin-*α* for 12 h and stained with DAPI. Bar = 20 *μ*M. Data is quantitated by cell counting. *N* = 4. (c) The protein expression of c-PARP and p-caspase 7. Cells were treated with 0.01% DMSO (CTL), 15 *μ*M AND with or without 15 *μ*M pifithrin, or pifithrin alone for 24 h, and cell lysates were analyzed for cleaved PARP (c-PARP) and c-caspase 7. *P < 0.05, **P < 0.01, as compared to the 0 h or CTL group, respectively. ^#^ *P* < 0.05, ^##^ *P* < 0.01, as compared to the AND-group. *N* = 4.

Procaspase 7 is cleaved to an active form, a heterotetramer of 2 large and 2 small subunits, by many enzymes, including caspases 3 and 9 [33, 36, 37]. In our study, caspases 3 and 9 were apparently not involved in AND-induced apoptosis, because these 2 caspases were not activated by AND treatment (Figure 2(a) and Supplementary Figure 3). The promoter region of caspase 7 is known to contain a binding site for p53 [38]. Further, p53 activation has been shown to lead to downstream activation of caspases 3 and 7, causing apoptosis in human glioblastoma cells [39]. First, we want to examine whether p53 is activated under AND treatment. After 24 h of AND treatment, the protein levels of both phosphorylated p53 and total p53 increased in a dose-dependent (Supplementary Figure 2(c)) and time-dependent (Figure 3(a)) manner. In Supplementary Figure 2(c), the phosphorylated p53 protein levels in C6 cells increased to 2.2-, 2.5-, and 4.1-fold following treatment with 10 *µ*M, 15 *µ*M, and 20 *µ*M AND, respectively, compared to treatment with DMSO, whereas the total p53 protein levels in C6 cells also increased to 2-, 2.1-, and 2.8-fold, respectively (Supplementary Figure 2(c)). As shown in Figure 5, the levels of phosphorylated p53 protein in C6 cells increased to 1.3-, 2.5-, and 3.2-fold following treatment with AND for 6 h, 12 h, and 24 h, respectively, relative to treatment for 0 h, whereas the total p53 protein levels in C6 cells also increased to 1.2-, 1.8-, and 2.8-fold (Figure 3(a)). To serve as a transcription factor, the activation of p53 included both phosphorylation and nuclear translocation. Immunofluorescent staining showed that p-p53 was expressed in the nucleus compared to control with AND treatment (Supplementary Figure 5). These results show that AND induced both the phosphorylation of p53 and p53 activation.

We then examined whether p53 plays a key role in AND-induced apoptosis. We pretreated C6 cells with a p53 inhibitor, pifithrin-*α*, and evaluated the extent of apoptotic cell death using DAPI stain (Figure 3(b)). The proportions of apoptotic cells were 5.0% ± 0.6% for the DMSO groups, 20.0% ± 2.0% for 15 *µ*M AND, and 7.5% ± 0.6% for 15 *µ*M AND plus pifithrin-*α* (Figure 3(b)). MTT and annexin V binding assays also showed that the effect of AND could be blocked by pifithrin-*α* (Supplementary Figure 4). Thus, AND induced apoptosis by p53 activation.

### 3.4. AND Induced Apoptosis of C6 Glioma Cells via the p53-Caspase 7-PARP Pathway

Because AND increased cellular p53 levels and the p53 inhibitor pifithrin-*α* reversed the effects of AND on apoptosis, we investigated the role of p53 in apoptosis. AND treatment led to increased levels of c-PARP, and pifithrin-*α* blocked this AND-induced PARP activation (Figure 3(c)). Further, AND treatment also led to increased levels of c-caspase 7, and pifithrin-*α* blocked this AND-induced caspase 7 activation (Figure 3(c)). The above findings suggest that AND can induce increased activation of p53 protein, which in turn activates the downstream caspase 7-PARP cascade.

### 3.5. Knockdown of p53 by siRNA Blocked AND-Induced Apoptosis

**Figure 4:**
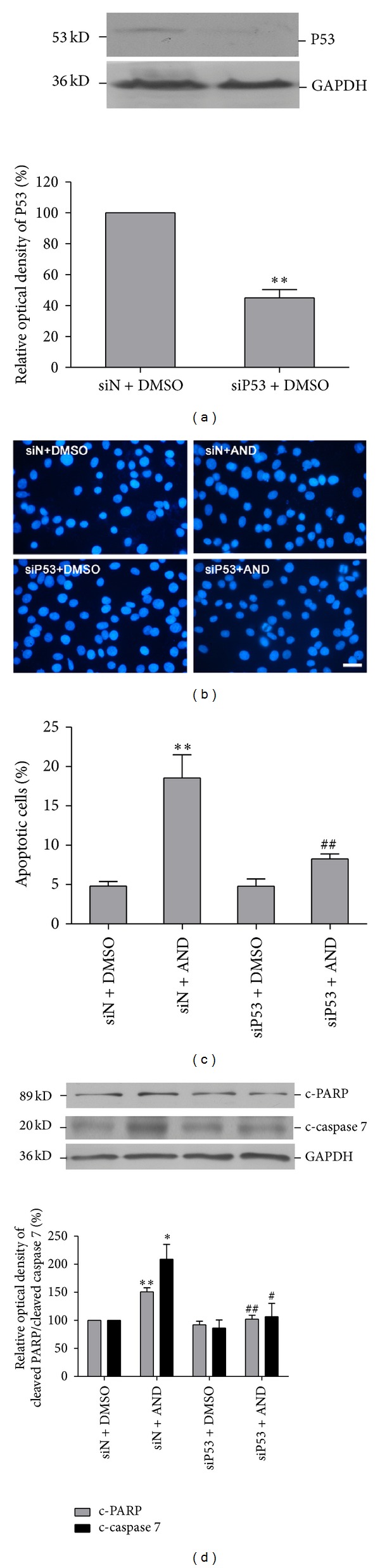
Effect of p53 siRNA on AND-induced apoptosis in C6 glioma cells. (a) Knockdown efficiency. Cells were transfected with p53 siRNA for 48 h, and cell lysates were analyzed for total p53 expression. *N* = 3. **P < 0.01, as compared to the siRNA-negative (siN) group. ((b)-(c)) Effect of p53 siRNA on AND-induced apoptosis. The cells were transfected with siN and siRNA-p53 (siP53) for 48 h and were then treated with 0.01% DMSO or 15 *μ*M AND for 12 h and stained with DAPI (b), and the ratio of apoptotic cells counted (c). *N* = 5. Bar = 20 *μ*m. **P < 0.01, compared to the siN + DMSO group. ^##^ *P* < 0.01 compared to the siN + AND group. (d) Cells were transfected with siN or siP53 for 48 h and then treated with 0.01% DMSO or 15 *μ*M AND. Cell lysates were analyzed for c-PARP and c-caspase 7. *N* = 3. *P < 0.05, **P < 0.01, as compared to the siN + DMSO. ^#^ *P* < 0.05, ^##^ *P* < 0.01, compared to the siN + AND group.

We further confirmed the role of p53 in AND-induced apoptosis by using RNA interference. A siRNA against p53 was introduced into C6 glioma cells, which decreased the level of total p53 protein to 55% compared to that in cells transfected with a negative siRNA (Figure 4(a)). After 12 h treatment, DAPI stain showed that the proportion of apoptotic cells was 4.8% ± 0.6% for cells treated with DMSO, 18.6% ± 2.9% for cells treated with 15 *µ*M AND, and 8.3% ± 0.6% for cells first transfected with p53 siRNA and then treated with 15 *µ*M AND (Figures 4(b) and 4(c)).

Since p53 siRNA reversed the apoptotic effect of AND, we examined how p53 siRNA affected the activation of PARP and caspase 7 by AND in C6 glioma cells. The levels of cleaved PARP and caspase 7 were elevated to 1.6- and 2.2-fold in negative siRNA groups following AND treatment for 24 h. In p53 siRNA-transfected cells, AND failed to activate caspase 7 and PARP (Figure 4(d)). This further supported the hypothesis that AND caused apoptosis of C6 glioma cells via the p53-caspase 7-PARP pathway.

### 3.6. Activation of p53 by AND Was Regulated by ERK

**Figure 5:**
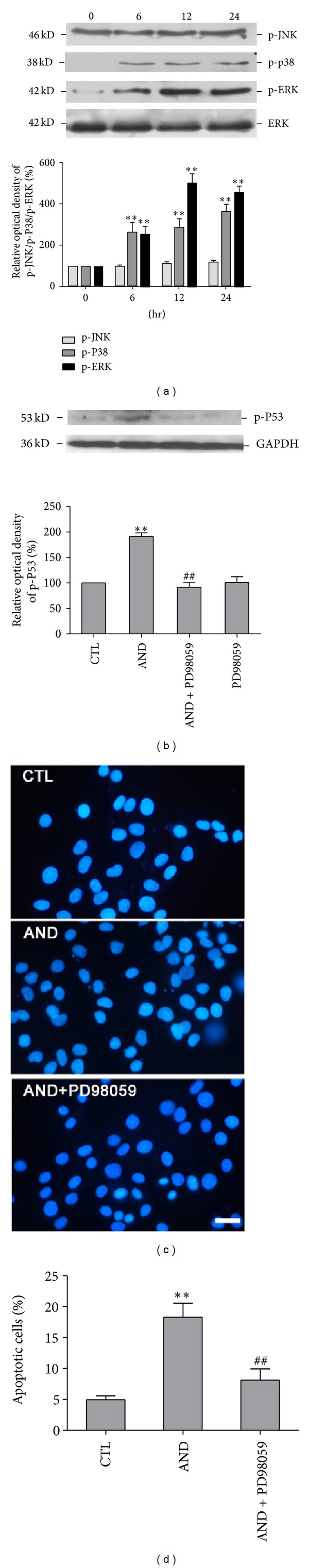
The expression of MAPK and the effect of MAPK inhibitors on AND-induced apoptosis in C6 glioma cells. (a) Time course study on MAPK activation. Cells were treated with 15 *μ*M AND for 0, 6, 12, or 24 h, and cell lysates were analyzed for p-JNK, pERK, p-38, or GADPH. The lower panel is the quantization of p-JNK, p-ERK, and p-p38 levels *P < 0.05, **P < 0.01, compared with the 0 h control. (b) The effect of ERK inhibitor on p53 phosphorylation. Cells were treated with 0.01% DMSO (CTL) or 15 *μ*M AND with or without 30 *μ*M PD98059 and were blotted for p-p53. *N* = 4. **P < 0.01, as compared to the CTL group. ^##^ *P* < 0.01, compared to the AND group. (c) The effect of ERK inhibitor on AND-induced cell death. Cells were treated with 0.01% DMSO (CTL) or 15 *μ*M AND with or without 30 *μ*M PD98059 and then were stained with DAPI. (d) Quantization of the apoptotic cell percentage. *N* = 4. **P < 0.01, compared to the DMSO group. ^##^ *P* < 0.01, as compared to the AND group.

ERK has been implicated in the regulation of p53 in the literature [40]. Following AND treatment, the levels of pERK and pP38 in C6 cells increased significantly in a time-dependent manner (Figure 5(a)), while the phosphorylation of JNK was not affected by the same treatment (Figure 5(a)). The pERK levels were elevated to 2.3-, 5-, and 4.5-fold after AND treatment for 6 h, 12 h, and 24 h, respectively (Figure 5(a)). Pretreatment of C6 cells with the ERK signaling inhibitor, PD98059, for 30 min, blocked the increased expression of p53 protein by AND (Figure 5(b)). Since inhibition of p38 kinase by SB203580 did not abrogate AND-induced p53 phosphorylation, we concluded that p38 kinase was not involved in this event (data not shown). Accordingly, p53 activation by AND was dependent on ERK signaling (Figure 5(b)).

To further confirm the role of ERK in C6 cell apoptosis triggered by AND, glioma cells were treated with an ERK signaling inhibitor, PD98059, for 30 min, followed by 15 *µ*M AND for 12 h. The apoptotic cell ratios were 8.3% ± 0.6% in AND groups pretreated with PD98059 and 18.3% ± 2.3% in AND-only groups (Figures 5(c) and 5(d)). MTT and annexin V binding assay also showed the blocking effect of AND (Supplementary Figure 6). Therefore, AND could induce apoptosis of C6 glioma cells via the ERK-p53-caspase 7-PARP signal transduction pathway.

**Figure 6:**
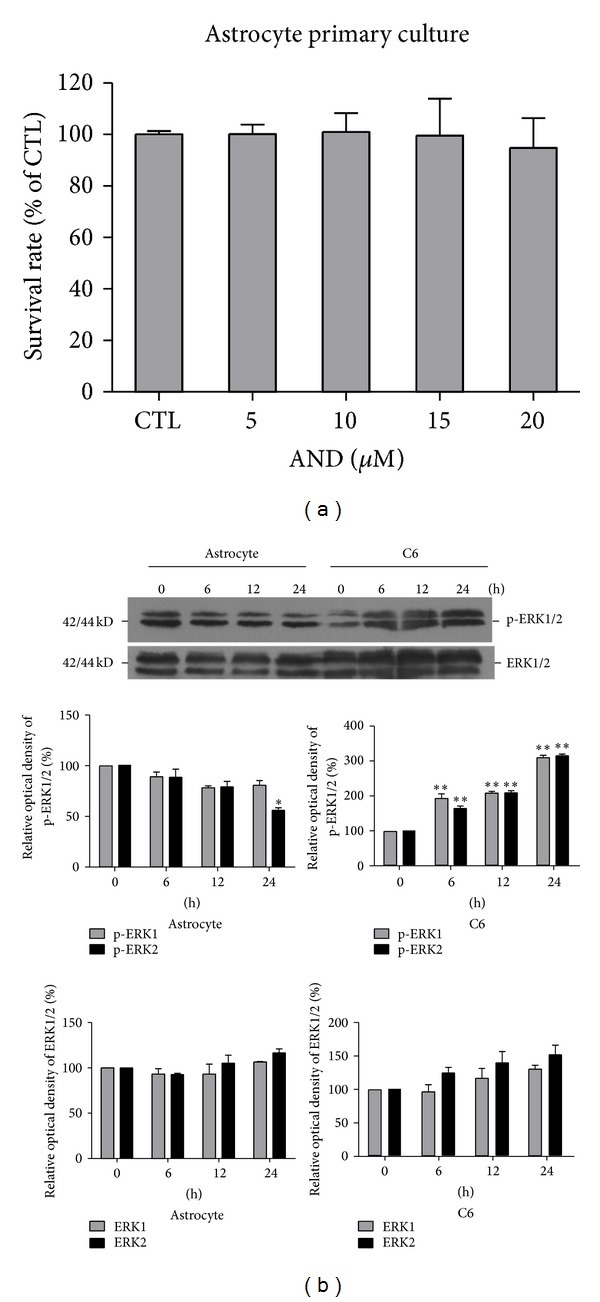
Effect of AND on cell viability and the expression of pERK in normal cultured rat astrocytes and C6 glioblastoma cells. (a) Cell survival analysis. Normal astrocytes were treated with 0.1% DMSO (CTL), 5, 10, 15, or 20 *μ*M of AND for 24 h, and the cell viability was determined by MTT assay. *N* = 3. (b) Blot analysis. Astrocytes and C6 cells were treated with 15 *μ*M AND for 0, 6, 12, or 24 h, and cell lysates were analyzed for pERK and ERK (upper panel). The quantization of p-ERK1, p-ERK2, ERK1, and ERK2 was presented in the following plots (lower panel). *N* = 3. *P < 0.05, **P < 0.01, as compared to the 0 h group.

We used normal astrocytes to compare the cytotoxicity of AND between normal cells and glioma cells. Cell viability was not affected by the presence of AND at various concentrations, ranging from 5 *µ*M to 20 *µ*M, compared to the control group (Figure 6(a)). Following treatment with 15 *µ*M AND for 24 h, the primary cultured astrocytes showed no increase of p53 or pERK protein levels (Figure 6(b)). This indicates that AND induces apoptosis, providing a tumoricidal effect, in C6 glioma cells.

**Figure 7:**
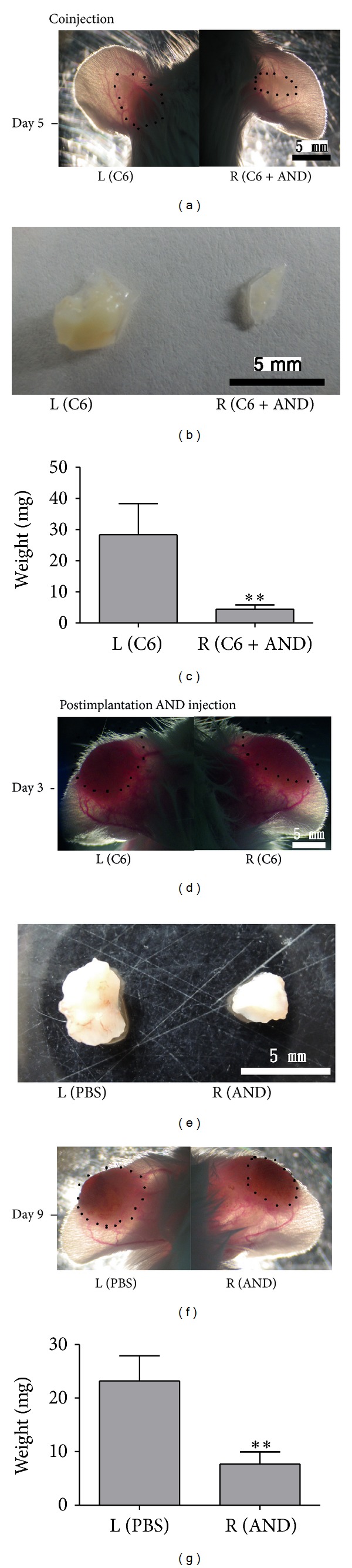
AND prevented the growth of C6 glioma* in vivo*. ((a)–(c)) The ears of ICR mice were injected with C6 cells with or without 20 AND *μ*M for 5 days. (a) An example of cell injection alone ear (C6) and AND plus cells injection ear (C6 + AND). (b) Tumors isolated from (a) at day 5. (c) Quantitation of tumor weights. *N* = 3, **P < 0.01 compared to the C6 group. ((d)–(g)) The ears of ICR mice were injected with C6 cells for 3 days (d) and then received injection with PBS (PBS) or 20 AND *μ*M (AND) twice at day 3 and day 6, and pictures were taken at day 3 (d) and day 9 (f). (e) Tumors isolated from (f) at day 9. (g) Quantitation of tumor weights. *N* = 3, **P < 0.01 compared to the PBS-group.

In order to further verify the effect of AND on tumor growth* in vivo*, two types of experiments were designed. In the first coinjection of AND way, C6 cells were injected subcutaneously into two ears with (right) or without (left) 20 *µ*M AND for 5 days (Figure 7(a)). AND treatment decreased the tumor weights by 86% (Figures 7(b) and 7(c)). In the second postimplantation AND injection of AND group, C6 cells were injected to both ears of ICR mice and allowed to grow for 3 days. At this stage, tumor masses on both sides appeared to be similar (Figure 7(d)). Then, PBS or 20 *µ*M AND were injected into the tumors of the left and right ear twice (at day 3 and day 6), respectively. AND treatment caused tumor regression as shown by 67% decrease of the tumor weight at day 9 (Figures 7(e), 7(f), and 7(g)).

## 4. Discussion

The poor prognosis of glioblastoma is due to therapeutic resistance and tumor recurrence after surgical removal. Treatment of high-grade gliomas is still only palliative. Studies have explored many techniques for glioblastoma treatments, including new chemotherapeutic agents such as camptothecin (CPT) [41], etoposide (VP) [42], emodin [43], and As_2_O_3_ [44]. This study used the C6 glioma cell line to evaluate the cytotoxic effects of AND and its potential therapeutic use. We have shown that AND effectively induced apoptosis in glioma cells via a novel signaling pathway, the ERK-P53-caspase 7-PARP pathway.

AND, the main constituent of* A. paniculata*, exhibits pharmacological effects on various cancers, including cell cycle arrest [45], autophagy [46], and apoptosis [24]. The effects of AND on cancer cells depend on the cell types and the concentrations applied. The concentrations used in previous studies were very wide, ranging from 0.7 to 100 *μ*M, and the concentration of AND we used in this study, 15 *μ*M, was within this range. AND only caused cell cycle arrest in hepatoma cells and in human glioblastoma [47, 48] but induced cell death in other cancer cells [24]. Interestingly, in this study, 15 *μ*M AND caused apoptosis in C6 glioma cells but had no effects on normal astrocytes (Figure 6), suggesting its potential use as a chemotherapeutic drug that has a selective cytotoxic effect on glioblastoma cancer cells.

In recent years, several studies focused on the apoptotic effect of AND on tumor cells. These studies found that AND-induced apoptosis occurred by the activation of proapoptotic JNK pathway [17, 26] and the suppression of antiapoptotic PI3K/AKT and ERK pathways [15, 19]. In addition, AND also triggered apoptosis through P53-induced caspase 3 activation [24]. In our system, we found that AND-induced apoptosis in C6 cells was mediated through the ERK-p53 pathway, since activation of p53 was decreased by an ERK1/2 inhibitor (Figure 5(b)). Although activation of ERK has been reported to be involved in AND-induced cell death in melanoma [24] and decreased invasion process in colon cancers [49], inhibition of ERK blocked the cytotoxic effect of AND in C6 cells (Figures 5(c) and 5(d)), suggesting that it is the upstream key regulator in AND-induced C6 cell death. ERK signaling, which was activated in AND-treated C6 cells, is an important signaling pathway involved in cell growth or apoptosis [50, 51]. The major differences in ERK signal activation in cell growth or cell death are the starting time and the duration of phosphorylation of ERK. In response to growth factor (EGF), ERK activation is rapid and transient, occurring within minutes of treatment [52]. We found that when cells were treated with AND, ERK was significantly activated and its phosphorylation remained high up to 24 h (Figure 5(a)). The same pattern of ERK activation has been observed in many anticancer drugs such as doxorubicin, quercetin [50], and paclitaxel [53]. p53, a tumor suppressor, is involved in the apoptotic effects of many drugs on cancer cells [54] and plays a central role in AND-induced apoptosis in C6 cells, as seen from the effect of a specific inhibitor (Figure 3) and siRNA (Figure 4). p53 is characterized as a stress-response protein, which is induced by DNA damage [55], oxidative stress [56], and deregulated oncogene expression [40]. Two major events are noticed in p53 activation. First, the half-life of the p53 protein is increased dramatically, which leads to p53 accumulation in stressed cells. Second, the phosphorylation and conformational change forces p53 to become a transcription factor. It has become clear that the p53 protein interacts functionally with the mitogen-activated protein kinase (MAPK) pathways, including JNK, the p38MAPK, and the ERK pathways. With stress exposure, MAPK phosphorylates and activates p53, leading to p53-mediated cellular responses [57]. Among the MAPK-mediated phosphorylations, ERK-mediated phosphorylation of p53 has been well observed in a number of experimental systems, including in ovarian cells induced by cisplatin [58] and in epidermal cell treated with resveratrol [59]. Our data correlates with these previous findings.

In many cancer cells, PARP is reported to be cleaved by activation of both caspases 3 and 7 during cell death induced by chemotherapeutic drugs, including camptothecin [60] and sorafenib [61]. It was also shown that caspase 7, which shares the same substrate preference as caspase 3, can cleave PARP more efficiently [62]. In our study, we were unable to detect caspase 3 by western blot when we induced cell death by AND in C6 cells (Figure 2(c)), and inhibition of caspase 7 prevented PARP cleavage (Figure 2(f)). These data suggested that PARP was cleaved by caspase 7 in our system. The same signaling was responsible for the apoptosis induced by *β*-lapachone in human prostate cancer cells [63] and by etoposide (VP16) phosphate in human leukemia cells [35].

Whether p53-induced activation of caspase 7 was due to a direct or indirect effect was a question that remained unanswered in this study. p53 is implicated in the induction of 2 distinct apoptotic signaling pathways—the intrinsic and extrinsic pathways. The extrinsic pathway involves death receptors, which lead to a caspase activation cascade, including caspase 8 and caspase 3. The intrinsic pathway is triggered by DNA damage and is associated with the release of cytochrome c from the intermembrane space of mitochondria into the cytoplasm. Cytochrome c forms a complex, termed the apoptosome, with apoptotic protease-activating factor 1 (APAF-1) and procaspase 9, and caspase 9 is activated to promote the activation of caspase 3, caspase 6, and caspase 7 [64, 65]. Both these pathways can trigger the activation of caspase 7 and PARP and lead cells to apoptosis. This study found that caspases 3 was not activated by AND (Figure 2(c)), and inhibition of caspase 9 by LEHD did not prevent AND-induced cell apoptosis of C6 cells (Supplementary Figure 3). Thus, caspase 9 was not involved in AND-induced caspase 7 activation. We believe that some regulatory signaling molecule(s), which may be caspase 8 in the intrinsic pathway, act between p53 and caspase 7. Despite being an intracellular signaling molecule, ERK also responds to stress, including oxidative stress [66] and ER stress [67]. Recent studies have suggested that both AND [68] and an AND derivatives (AL-1) [69] exert cytotoxic effects on cells through a ROS-dependent mechanism. We also demonstrated that ROS is involved in AND-induced apoptosis in C6 cells by ROS chelators, NAC, and DTT (Supplementary Figure 7) with MTT and annexin V binding assay. Thus, further studies should explore whether ROS activates ERK signaling, as well as the underlying mechanisms.

## 5. Conclusion

In conclusion, AND exerts its cytotoxicity on C6 glioma cells through the ERK-p53-caspase 7-PARP apoptotic pathway. AND treatment inhibited the tumor growth in coinjection experiment and caused the regression of the tumors in postinjection experiment. This regression of well-formed tumors was mediated by AND-induced cell death. The successful application of AND on animal models strengthens its clinical use in cancer therapy. Because of the selective toxicity to only glioma cells, and not to normal astrocytes, AND has great potential to be an anticancer drug.

## Supplementary Material

AND and DAPI were purchased from Sigma. DEVD, 3AB, DTT, and NAC were purchased from BioMol. pifithrin-*α* is purchased from Enzo. LEHD is purchased from Biovision. PD98059 was purchased from Cell Signaling Technology. Annexin V is from the Aoptosis Detection kit (Strong Biotech Corporation, AV050, Taipei, Taiwan). c-caspase 7, c-PARP, p-P53, and P53 antibodies were purchase from Cell Signaling Technology. GAPDH was purchased from GeneTex.
