# Supplementary material for: Andrographolide Induces Apoptosis of C6 Glioma Cells via the ERK-p53-Caspase 7-PARP Pathway
Source: Biomed Res Int. 2014 Aug 5;2014:312847. doi: 10.1155/2014/312847 (PMC4139087; doi:10.1155/2014/312847)
Supplement: Supplementary file 1 — AND and DAPI were purchased from Sigma. DEVD, 3AB, DTT, and NAC were purchased from BioMol. pifithrin-α is purchased from Enzo. LEHD is purchased from Biovision. PD98059 was purchased from Cell Signaling Technology. Annexin V is from the Aoptosis Detection kit (Strong Biotech Corporation, AV050, Taipei, Taiwan). c-caspase 7, c-PARP, p-P53, and P53 antibodies were purchase from Cell Signaling Technology. GAPDH was purchased from GeneTex. [file 312847.f1.pdf]

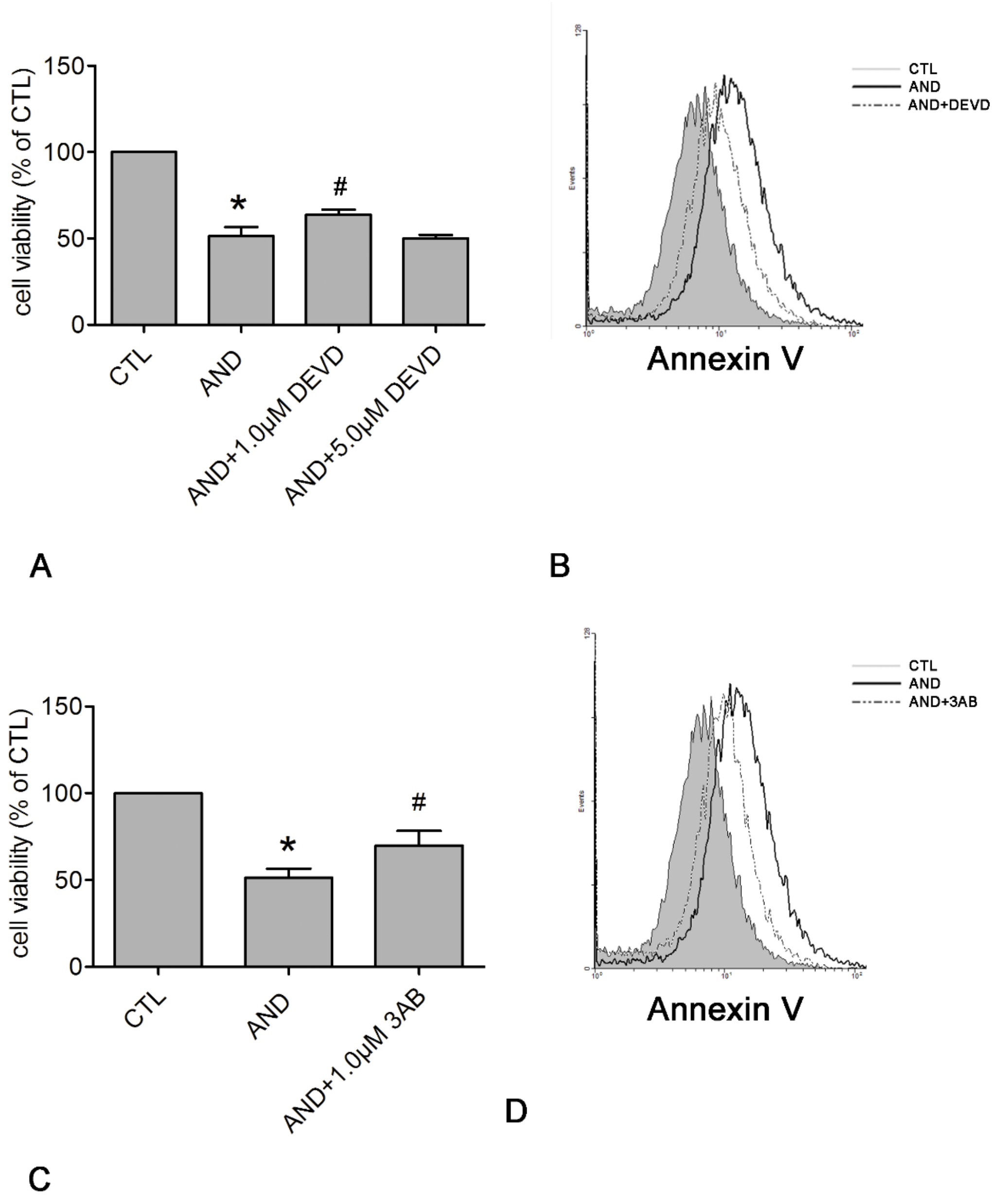

supplementary figure 1

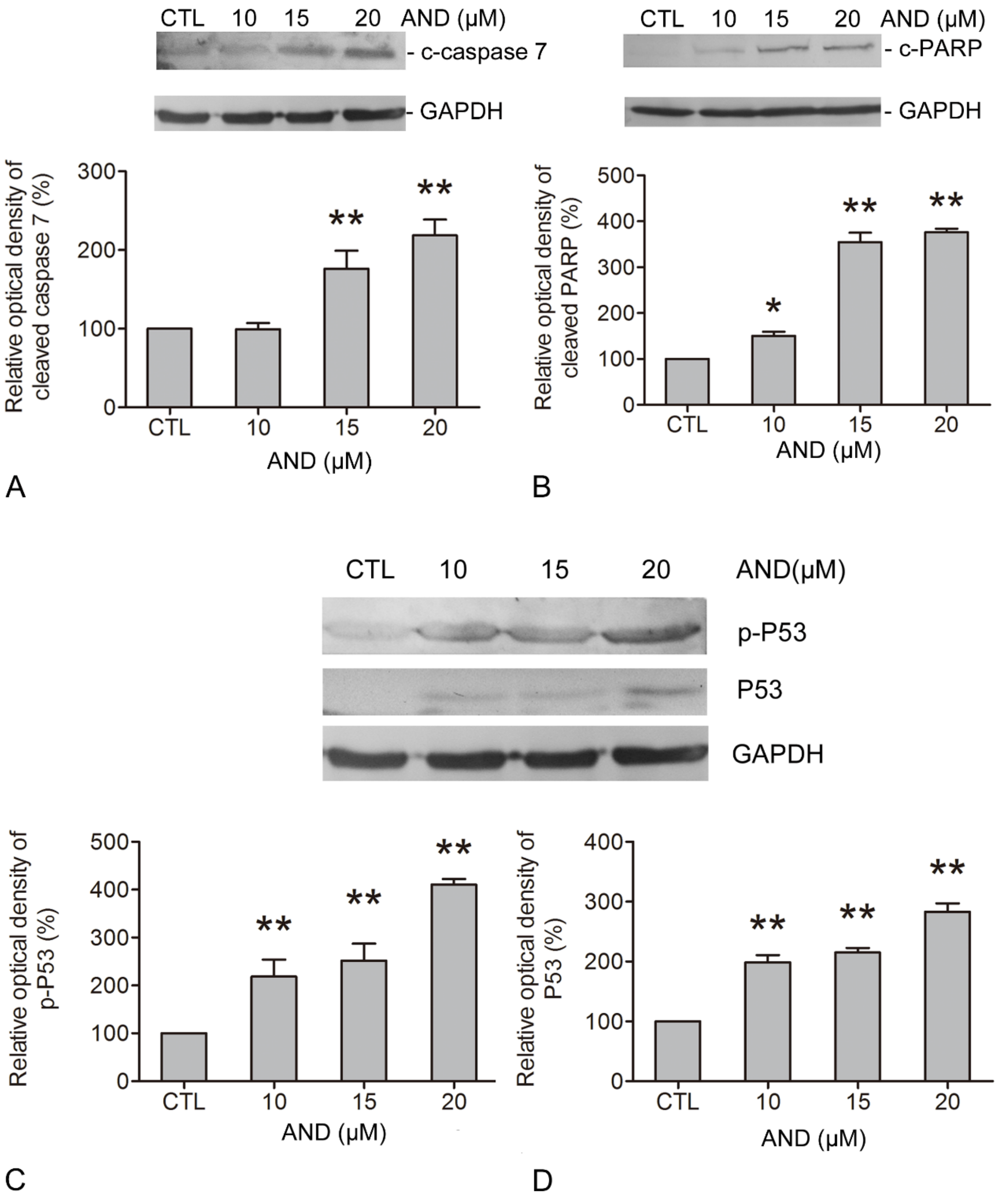

supplementary figure 2

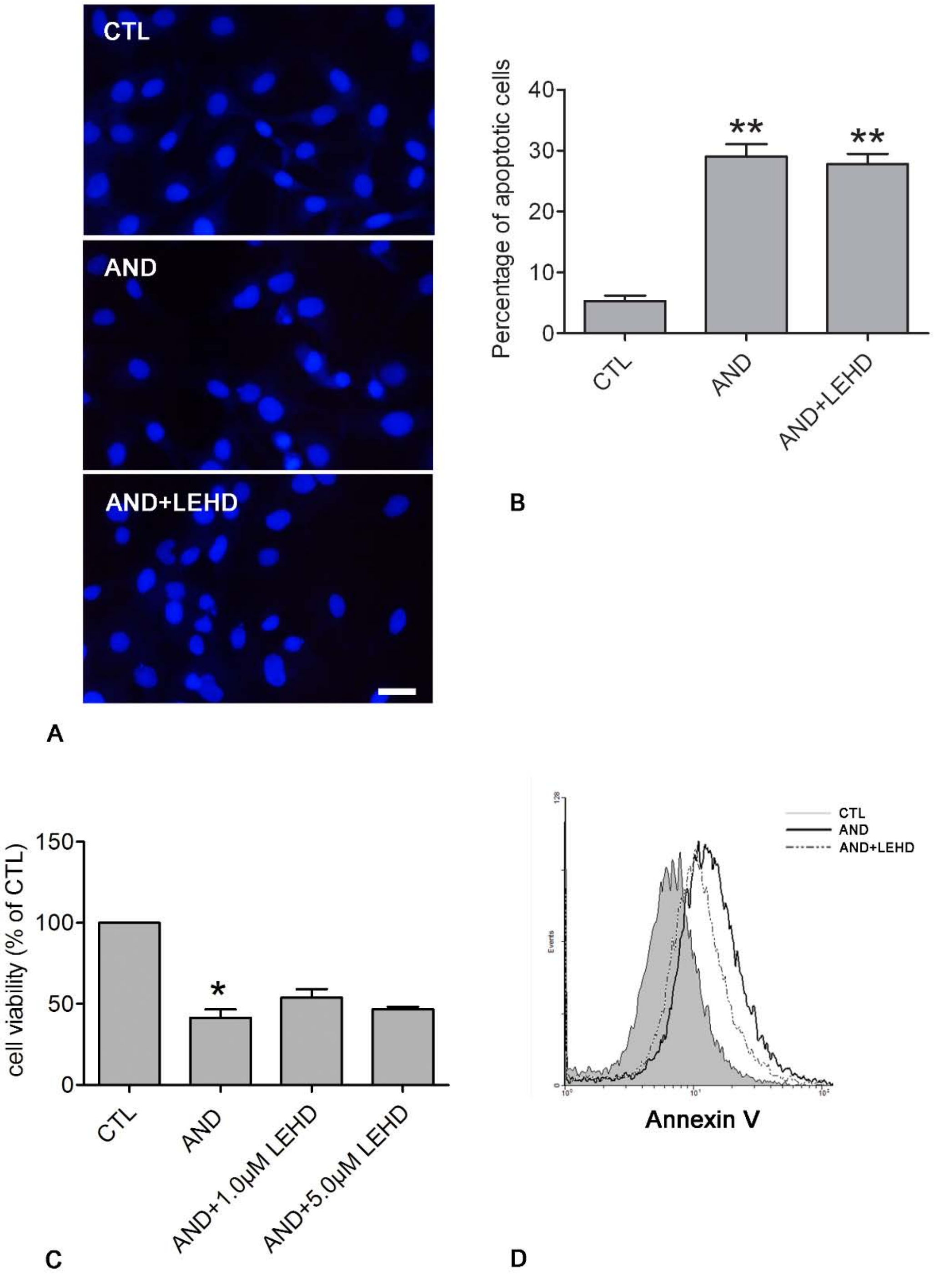

supplementary figure 3

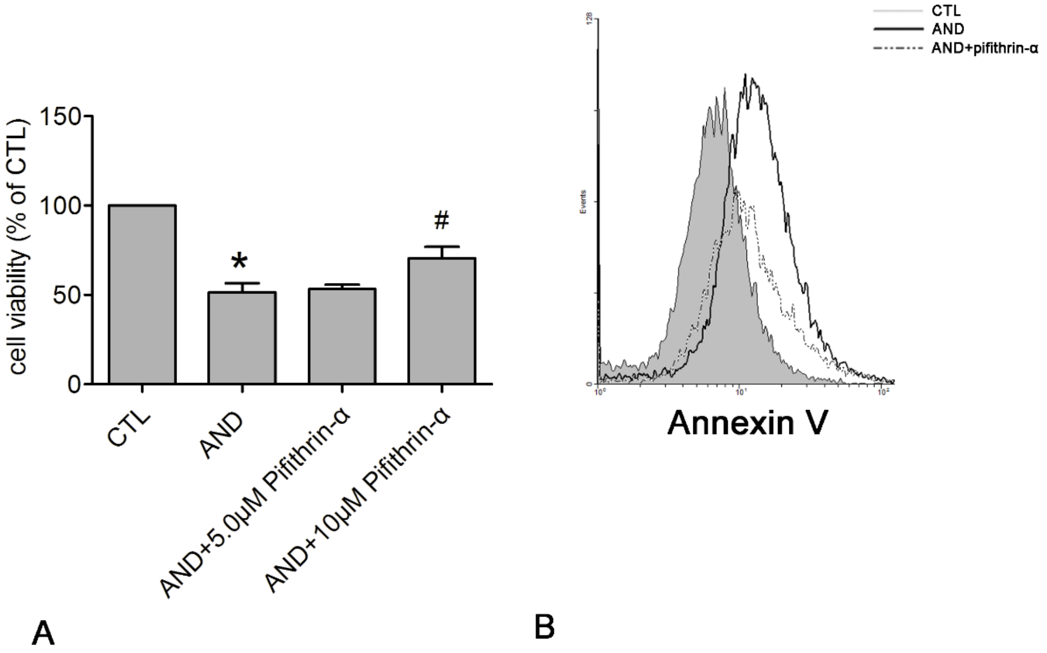

supplementary figure 4

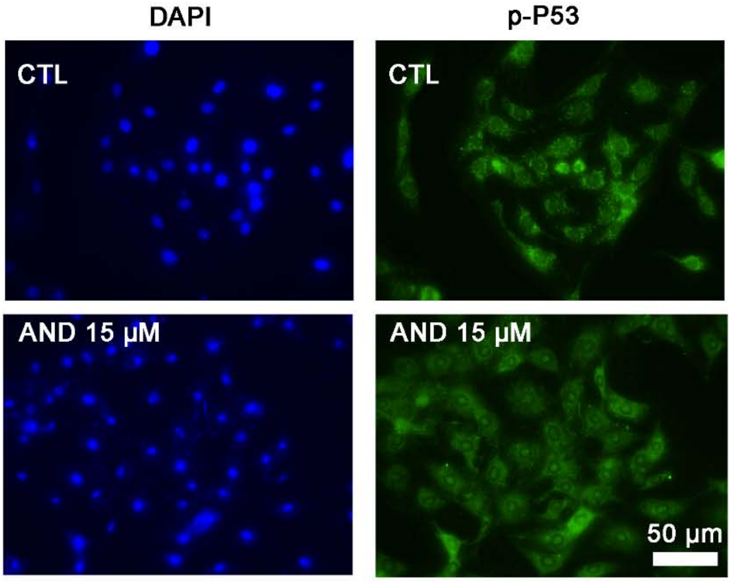

A

supplementary figure 5

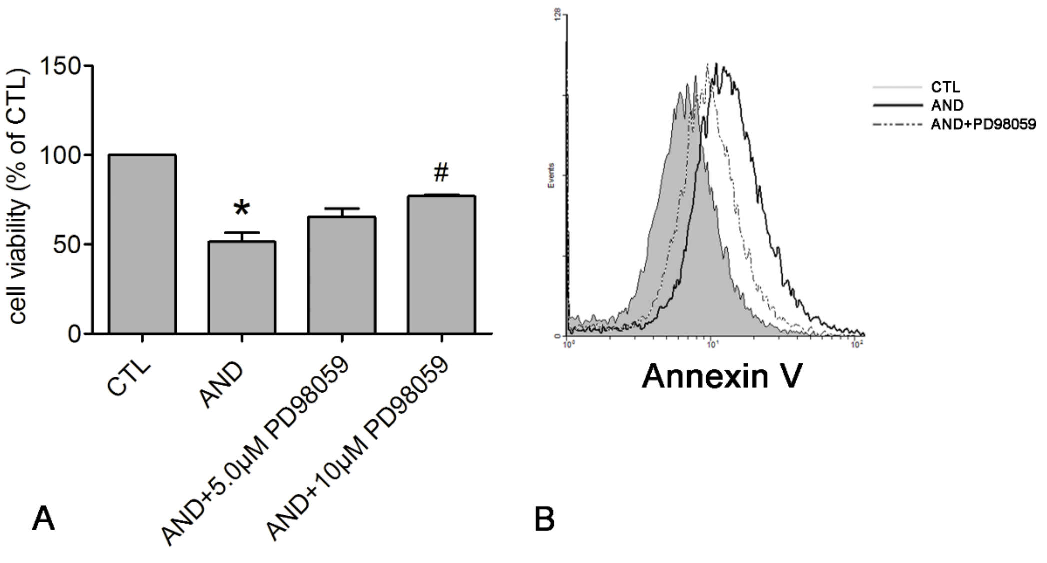

supplementary figure 6

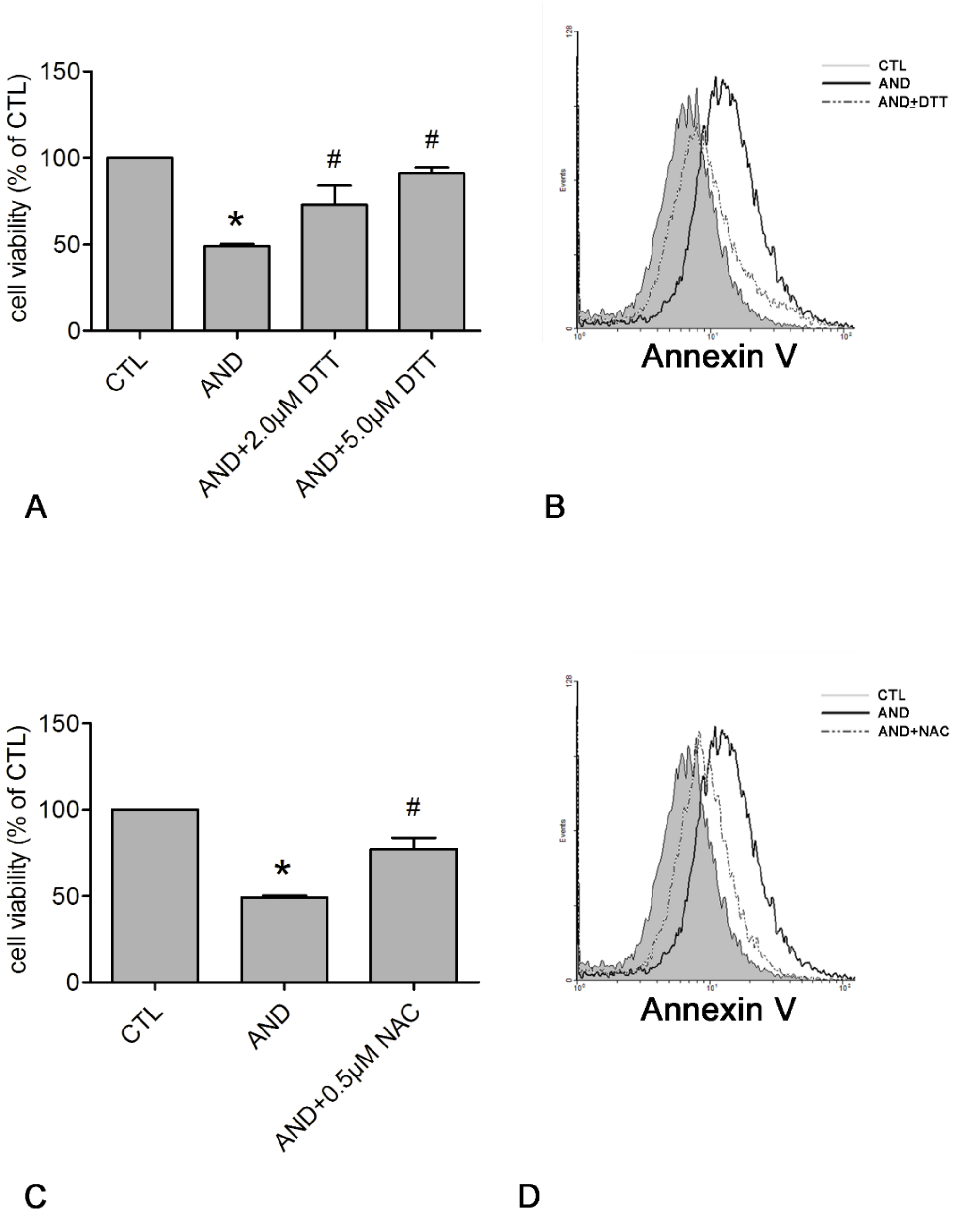

supplementary figure 7

Materials and Methods for Supplementary Figures

Drug

ROS chelators, DTT and NAC, were purchased from Sigma.

Western blotting

Cells were treated with 0.1% DMSO (CTL) or 10, 15, or 20 μM AND for 24 h. Proteins were extracted from cells and analyzed by western blotting for cleaved caspase 7 (c-caspase 7), cleaved PARP (c-PARP), phosphorylated p53 (p-p53), p53, and GAPDH (internal control). Band densities on the membrane were quantified by densitometry by using Gel Pro 3.1 (Media Cybernetics, Silver Spring, MD, USA), setting the band density of the control sample as 100% and expressing the band densities of the test samples as percentages of this.

Apoptosis Assay

DAPI stain was used to detect apoptotic cells. Cells were seeded on coverslips. After various treatments, cells were washed with ice-cold PBS and stained for 15 min with 1 μg/mL of DAPI in 0.9% NaCl. Coverslips were mounted onto slides using fluorescence mounting medium (70% glycerol and 2% propyl gallate in PBS). Cell images were captured with fluorescence microscopy and digital camera.

Annexin V binding assay

For detection of apoptosis, cells were treated with AND for 0–24 h and then trypsinized. After washes with cold PBS, the cells were stained with annexin-V-FITC (Strong Biotech Corporation, Taipei, Taiwan) for 15 min and analyzed by flow cytometry.

Immunofluorescence Staining.

Cells were incubated with 15 μM AND for 12h and were then fixed in 4% paraformaldehyde for 15 min at room temperature (RT). After being blocked for 1 h at RT with 10% normal goat serum (NGS) containing 0.05% Triton X-100, the cells were stained overnight at 4°C with monoclonal antibody against p-p53 (1:1,000), incubated with Dylight-conjugated secondary antibody and Hoechst dye for 1 h at RT, and examined and photographed using a Leica fluorescence microscope.
